# Association of live microbes intake and risk of all-cause, cardiovascular disease, and cancer-related mortality in patients with chronic kidney disease

**DOI:** 10.1080/0886022X.2024.2449196

**Published:** 2025-01-06

**Authors:** Debin Chen, Yongju Ye, Yining Li, Erxu Xue, Qijun Zhang, Youlan Chen, Jianhui Zhao

**Affiliations:** a Chronic and Endemic Disease Prevention and Control Division, Xiamen Center for Disease Control and Prevention, Xiamen, China; b Department of Gynaecology, Lishui Hospital of Traditional Chinese Medicine, Lishui, China; c Department of Radiology, The Second Xiangya Hospital, Central South University, Changsha, China; d Nursing Department, Sir Run Run Shaw Hospital, Zhejiang University School of Medicine, Hangzhou, China; e Cardiovascular Department, The Affiliated People’s Hospital of Ningbo University, Ningbo, China; f Department of Epidemiology and Health Statistics, School of Public Health, Zhejiang University School of Medicine, Hangzhou, China

**Keywords:** Chronic kidney disease, live microbes, all-cause mortality, cardiovascular disease mortality, cancer-related mortality

## Abstract

**Background:**

Chronic kidney disease (CKD) is a prevalent chronic, non-communicable disease. The long-term health effects of dietary live microbes, primarily probiotics, on CKD patients remain insufficiently understood. This study aims to investigate the association between dietary intake of live microbes and long-term health outcomes among individuals with CKD.

**Methods:**

Utilizing the National Health and Nutrition Examination Survey (NHANES) database, Cox regression analysis assessed the association between medium and high categories dietary live microbe intake and health outcomes (all-cause, cardiovascular disease [CVD], and cancer-related mortality) in CKD patients.

**Results:**

A total of 3,646 CKD patients were enrolled. During the follow-up period, 1,593 all-cause mortality events were recorded, including 478 CVD deaths and 268 cancer deaths. In the fully adjusted model, compared to CKD patients in the lowest quartile (quartile 1) of live microbes intake, those in quartiles 3 and 4 exhibited a 20% and 26% reduced risk of all-cause mortality, with hazard ratios (HR) of 0.80 (95% confidence interval, CI: 0.69, 0.94) and 0.74 (95% CI: 0.62, 0.90), respectively. Additionally, compared to those with low live microbe intake (quartile 1), higher live microbe intake in quartile 4 was associated with a 37% reduction in the risk of CVD mortality for CKD patients, with an HR of 0.63 (95% CI: 0.45, 0.88). Consistent results were observed in subgroup and sensitivity analyses. A significant negative association was observed between live microbe intake and the risk of all-cause mortality as well as CVD mortality in the CKD population, with a p-value for trend < 0.05.

**Conclusion:**

Our study indicated that high dietary live microbe intake could mitigate the risk of all-cause and CVD mortality in CKD patients. These findings support the inclusion of live microbes in dietary recommendations, highlighting their significant roles in CKD.

## Introduction

Chronic kidney disease (CKD) is a chronic non-communicable disease characterized by positive urine protein, abnormal urine sediment or decreased glomerular filtration rate, and abnormal renal structure or function lasting for more than three months [1]. Chronic kidney disease, recognized as a significant chronic non-communicable disease, has imposed a substantial burden on global public health over the past few decades. The risk factors of CKD include proteinuria, diabetes, hypertension, drugs and dietary behavior factors. Among them, dietary behavioral factors play an important role in the occurrence, development, and prognosis of CKD [2–4].

Dietary live microbes are substance present in daily human diet, particularly in yogurt, fermented foods, and unpeeled vegetables and fruits [5]. Some current studies have found that microbes changes in the human gut and external environment play an important role in regulating the occurrence and development of some chronic diseases [6–8]. The main edible strains are *Lactobacillus strains* and *Bifidobacterium strains. Lactobacillus acidophilus* helps with digestion, improves lactose intolerance, and enhances immune function [9]. *Lactobacillus rhamnosus* can help control diarrhea associated with antibiotics and enhance immunity [10]. Furthermore, *Bifidobacterium longum* and *Bifidobacterium brevis* exhibit notable functions in alleviating and improving symptoms of irritable bowel syndrome and demonstrating antioxidant activity, respectively [11, 12].

Maria et al. calculated the live microbe content in these foods and categorized them into three distinct types based on their levels of live microbes, using data from the National Health and Nutrition Examination Survey (NHANES) [5]. Among them, low grade (<10^4^ CFU/g) foods mainly included pasteurized foods, medium grade (10^4^-10^7^ CFU/g) foods included non-peeled fresh fruits and vegetables, and high grade (>10^7^ CFU/g) foods included unpasteurized or fermented foods and probiotic supplements. Dietary live microbes primarily coexist with the human intestinal microenvironment. By promoting homeostasis of the intestinal microbiota and inhibiting harmful bacteria, these microbes may prevent the colonization of exogenous pathogenic bacteria within the gastrointestinal tract. And the metabolites produced by the colonization of microbes in the gastrointestinal tract will also neutralize and inhibit the production of harmful bacterial toxins, reducing local and systemic inflammatory reactions in the body. Current studies have found that live microbes have positive impacts on chronic diseases such as abdominal aortic calcification [6], diabetic kidney disease [13] and elderly cognition [14]. Considering that dietary intake of live microbes may play a crucial role in modulating the body’s acid-base balance and systemic inflammatory responses during the progression of chronic kidney disease, dietary live microbes could potentially improve adverse outcomes in CKD patients.

Herein, we conducted a cohort analysis to examine the relationship between dietary intake of live microbes and the risk of all-cause, cardiovascular disease (CVD), and cancer-related mortality among CKD patients residing in the United States.

## Materials and methods

### Data source and participants

**Figure 1.**
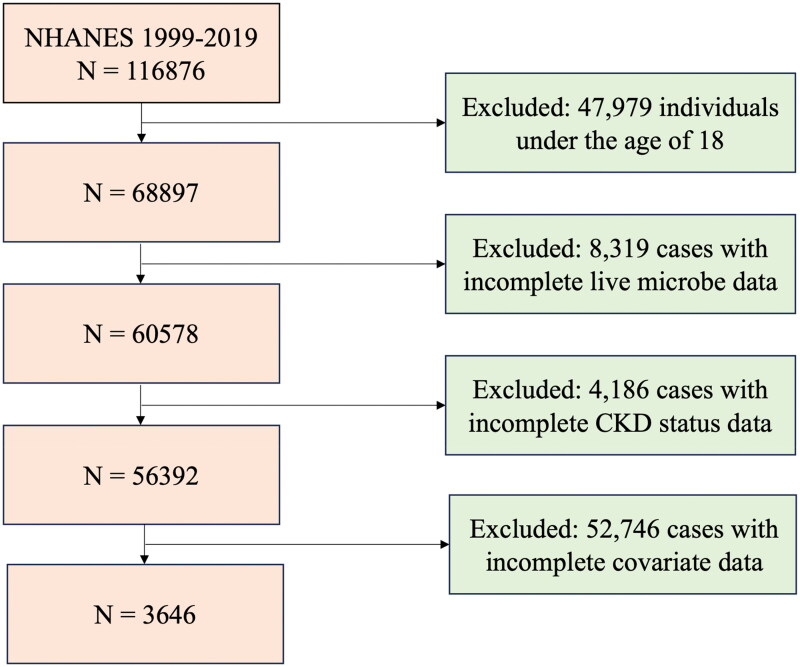
Flowchart of participant inclusion. Abbreviation: CKD, Chronic Kidney Disease.

Data was sourced from NHANES, a large-scale survey of U.S. residents conducted using complex multi-stage probability sampling. We collected demographic information, physical examination results, laboratory indicators, dietary intake, and other health data of U.S. residents every two years. The data collection protocol and procedures were approved by the National Center for Health Statistics (NCHS) Institutional Review Board, and all participants provided informed consent before the collection of personal information and laboratory samples. We selected survey data from the 1999 to 2018 NHANES dataset for analysis. After excluding missing data on live microbe intake, covariates, and CKD diagnostic indicators, 3,646 CKD patients were included in the analysis (Figure 1).

### Estimation of live microbe intake

The NHANES study collected dietary consumption data from participants using a 24-h dietary recall survey. The nutritional content of the foods and beverages reported was sourced from the nutrient database of the U.S. Department of Agriculture (USDA), which includes detailed information on macronutrients, trace elements, and phyto-active ingredients [15]. Four experts classified the food based on the distribution and content of live microbes (including bacteria and fungi) [5]. The classification principles are mainly based on statistical values [16–19] and authoritative reviews in previous literatures [20], and finally determine the food classifications with different live microbes contents: low (<10^4^ CFU/g foods), medium (Med,10^4^-10^7^ CFU/g foods) and high (Hi, >10^7^ CFU/g foods). In our study, we primarily focused on the relationship between the intake of medium (Med) and high (Hi) categories of live microbes and the long-term health outcomes in CKD patients.

### Outcome

The follow-up time, survival status, and outcome data of CKD patients in this survey were determined using the National Death Index (NDI) [21], all information about the participants above was until 31 December 2019. The outcomes of all-cause mortality, as well as CVD mortality and cancer mortality among CKD patients, were determined using the International Classification of Diseases (ICD). Specifically, CVD mortality was defined as death caused by heart-related diseases (I00-I09, I11, I13, and I20-I51), while cancer mortality was defined as death caused by malignant tumors (C00-C97). The follow-up duration was calculated from the date of inclusion in the study until the endpoint of death.

### Covariates

Given the previous analysis on the association between dietary live microbes and other diseases [6,14] and the daily behavioral habits of American people, the potential confounding factors that might affect the association between live microbes and CKD were included in this study. The including covariates were as follows: age (years old), gender (male, female), ethnicity (Mexican American, Non-Hispanic black, Non-Hispanic white and others), the income level was measured by poverty income ratio (PIR, the ratio of family income divided by a poverty threshold specific for family size using guidelines from the US Department of Health and Human Services) and categorized as ≤ 1.0, 1.1-3.0, and > 3.0 [22]. Marital status (married/living with partner, never married, widowed/divorced/separated); education levels (below high school, high school, above high school); smoking status was categorized as never (< 100 cigarettes in lifetime), former (≥ 100 cigarettes and had since quit) and now (> 100 cigarettes in lifetime and smoked on some days or even daily); individual drinking status was categorized as: never (<12 drinks in lifetime), former (≥ 12 drinks in one year), mild (≤ 1 drink per day for female and ≤ 2 drinks for male), moderate (2 drinks per day for women and 3 drinks per day for male, or > 2 binge drinking and < 5 times per month), heavy (≥ 3 drinks per day for female and ≥ 4 drinks per day for male, or binge drinking ≥ 5 per month) [23]; body mass index (BMI) was categorized as: < 25.0, 25.0-29.9 and ≥ 29.9; usage of prebiotics and probiotics (yes/no). Physical activity: calculated by Metabolic Equivalent of Task (MET)-min per week, 1 week of total MET-minutes was the sum of work activity (days vigorous work × minutes vigorous-intensity work × 8, days moderate work × minutes moderate-intensity work × 4), recreational activities (days vigorous recreational activities × minutes vigorous recreational activities × 8, days moderate recreational activities × minutes moderate recreational activities × 4), and walk or bicycle (number of days walk or bicycle × minutes of walk or bicycle for transportation × 4), and then was divided into three quantiles (low, middle, high) [24]; hypertension (diagnosed by any of the following: use of antihypertensive drugs, brachial artery pressure >140 mmHg/> 90 mmHg); diabetes (diagnosed by a doctor or health professional, use of hypoglycemic drugs, glycated hemoglobin (HbA1c) concentration > 6.5%, fasting blood glucose >7.0 mmol/L, random blood glucose > 11.1 mmol/L or oral glucose tolerance test [OGTT] > 11.1 mmol/L); given the impact of dietary behavioral factors of residents, then healthy eating index-2015 (HEI-2015) was included in study as a covariance [25]. The laboratory testing indicators included alanine aminotransferase (ALT), aspartate aminotransferase (AST), serum creatinine, glycated hemoglobin (HbA1c), serum uric acid, and triglycerides.

### Statistical analysis

All survey statistical results were analyzed using appropriate sampling weights. Continuous variables were presented as weighted means ± standard deviations, and categorical variables as proportions. Statistical differences were compared using weighted t-tests and chi-square tests. Weighted Cox regression analysis to adjust the hazard ratio (HR) and 95% confidence interval (CI). Three models were established: Crude model: no covariate adjusted; Model 1, adjusted for age, gender, ethnicity, education level, marital status, and PIR; Model 2, further adjusted for BMI, smoking status, drinking status, HEI-2015 scores, metabolic equivalents of task (METs), laboratory test indicators, and history of chronic disease.

The trend tests and interaction analysis between covariates and the live microbes were condected. Also, we conducted the following sensitivity analyses: First, we excluded participants who died within the first two years of follow-up; second, we excluded participants who consumed non-food prebiotics and probiotics supplements. Finally, we excluded the utilization of antibiotics within the past year. A bilateral p-value of < 0.05 was considered statistically significant, all statistical analyses were performed using R software version 4.2.3 [26].

## Results

Basic demographic and daily behaviors characteristics of the 3,646 participants, categorized according to different levels of live microbe intake, were presented in Tables 1 and 2. The average age of the surveyed population was 63.86 ± 0.39 years, with 44.65% being male and 55.35% female, and 61.87% identified as non-Hispanic white. Compared to the low dietary live microbial intake group, the high intake group generally had higher levels of education, PIR, and lower levels of smoking, binge drinking. The clinical markers and chronic diseases characteristics were presented in Table 2. The high intake group was associated with lower levels of hypertension, serum creatinine, and eGFR and higher levels of HEI-2015 scores and energy intake (all *p* < 0.05). The distribution of anti-chronic disease drug use in CKD patients was showed in Supplementary Table 1.

**Table 1.** Demographic and clinical characteristics of chronic kidney disease patients.

Characteristic	Overall (*n* = 3,646)	Dietary intake of MedHi live microbes^a^				P value
		Quartile 1	Quartile 2	Quartile 3	Quartile 4	
Age (years)	63.86 ± 0.39	63.24 ± 0.66	64.41 ± 0.77	65.03 ± 0.68	63.06 ± 0.67	0.11
Gender, n (%)						0.18
Female	2,018 (55.35)	634 (58.07)	468 (62.83)	465 (62.99)	451 (59.46)	
Male	1,628 (44.65)	589 (41.93)	342 (37.17)	340 (37.01)	357 (40.54)	
Ethnicity, n (%)						<0.0001
Non-Hispanic white	2,256 (61.87)	671 (72.98)	504 (80.18)	522 (80.94)	559 (85.99)	
Mexican American	423 (11.60)	139 (4.92)	108 (4.72)	89 (3.94)	87 (3.24)	
Non-Hispanic black	594 16.29)	284 (12.63)	124 (8.06)	101 (6.07)	85 (4.92)	
Others	373 (10.24)	129 (9.47)	74 (7.04)	93 (9.06)	77 (5.85)	
Marital status, n (%)						<0.001
Married/living with partner	2,053 (56.30)	636 (55.00)	461 (60.13)	483 (64.43)	473 (65.33)	
Never married	247 (6.77)	105 (8.82)	40 (5.06)	46 (5.82)	56 (6.67)	
Widowed/divorced/separated	1,346 (36.93)	482 (36.18)	309 (34.81)	276 (29.75)	279 (28.00)	
Education, n (%)						<0.0001
Below high school	645 (17.69)	270 (14.92)	156 (11.77)	124 (10.46)	95 (7.70)	
High school	1,258 (34.50)	451 (36.97)	279 (37.64)	269 (30.55)	259 (27.84)	
Above high school	1,743 (47.81)	502 (48.10)	375 (50.59)	412 (58.99)	454 (64.46)	
Poverty income ratio, n (%)						<0.0001
≤1	566 (15.52)	229 (13.90)	140 (11.67)	103 (8.43)	94 (8.43)	
1.1-3.0	1,776 (48.71)	631 (47.30)	401 (46.74)	397 (43.40)	347 (34.24)	
> 3.0	1,304 (35.77)	363 (38.80)	269 (41.59)	305 (48.17)	367 (57.33)	
Smoking status, n (%)						<0.0001
Never	1,807 (49.56)	571 (33.94)	393 (34.56)	422 (36.33)	421 (40.36)	
Former	1,373 (37.66)	433 (46.95)	307 (50.48)	306 (51.98)	327 (52.14)	
Now	466 (12.78)	219 (19.11)	110 (14.96)	77 (11.69)	60 (7.50)	
Drinking status, n (%)						0.04
Never	611 (16.76)	198 (15.15)	137 (15.88)	148 (16.53)	128 (13.07)	
Former	1,087 (29.81)	412 (28.69)	247 (26.77)	215 (24.60)	213 (22.88)	
Mild	1,277 (35.02)	379 (32.05)	273 (35.69)	308 (38.87)	317 (41.19)	
Moderate	362 (9.93)	120 (13.00)	67 (10.67)	83 (13.10)	92 (14.49)	
Heavy	309 (8.48)	114 (11.11)	86 (11.00)	51 (6.90)	58 (8.37)	
Body mass index (kg/m^2^), n (%)						0.27
< 25.0	944 (25.89)	315 (26.21)	206 (27.36)	203 (24.30)	220 (30.29)	
25.0-29.9	1,236 (33.90)	398 (32.10)	274 (30.48)	285 (35.27)	279 (32.54)	
> 29.9	1,466 (40.21)	510 (41.69)	330 (42.16)	317 (40.43)	309 (37.17)	
Physical activity (MET), n (%)						0.01
Low (<120.89)	1,716 (47.07)	629 (47.22)	398 (44.43)	372 (44.80)	317 (34.23)	
Middle (120.89-1272.13)	1,044 (28.63)	322 (28.99)	215 (30.52)	231 (30.39)	276 (36.34)	
High (>1272.13)	886 (24.30)	272 (23.79)	197 (25.05)	202 (24.81)	215 (29.43)	

^a^The results are presented as [mean ± standard deviation, number (percentage)].

Abbreviation: CKD: Chronic Kidney Disease; PIR: poverty-income ratio; MedHi: medium and high live microbes; MET: metabolic equivalent of task.

**Table 2.** Clinical markers and comorbid conditions in chronic kidney disease patients^a^.

Characteristics	Overall (*n* = 3,646)	Dietary intake of MedHi live microbes				P value
		Quartile 1	Quartile 2	Quartile 3	Quartile 4	
Diabetes, n (%)						0.35
Yes	1,306 (35.82)	459 (32.46)	301(31.68)	268 (30.28)	278 (27.72)	
No	2,340 (64.18)	764 (67.54)	509 (68.32)	537 (69.72)	530 (72.28)	
Hypertension, n (%)						<0.001
Yes	2,711 (74.36)	908 (70.14)	601 (72.13)	633 (74.53)	569 (62.43)	
No	935 (25.64)	315 (29.86)	209 (27.87)	172 (25.47)	239 (37.57)	
Coronary heart disease, n (%)						0.28
Yes	459 (12.59)	159 (11.21)	81 (8.97)	108 (12.05)	111 (12.57)	
No	3,187 (87.41)	1,064 (88.79)	729 (91.03)	697 (87.95)	697 (87.43)	
Non-food prebiotic, n (%)						0.80
Yes	103 (2.83)	33 (3.33)	18 (2.99)	28 (4.28)	24 (3.65)	
No	3,543 (97.17)	1,190 (96.67)	792 (97.01)	777 (95.72)	784 (96.35)	
Non-food probiotic, n (%)						0.16
Yes	84(2.30)	22 (2.67)	13 (2.15)	21 (4.08)	28 (4.52)	
No	3,562 (97.70)	1,201 (97.33)	797 (97.85)	784 (95.92)	780 (95.48)	
Energy intake, kcal/d	3586.23 ± 31.52	3480.35 ± 59.10	3476.76 ± 71.21	3560.60 ± 57.36	3824.99 ± 60.89	<0.001
Healthy eating index-2015 score, n (%)						<0.0001
Quartile 1 (0-48.48)	1,163 (31.90)	547 (46.24)	293 (38.79)	206 (28.81)	117 (14.23)	
Quartile 2 (48.48-60.68)	1,245 (34.15)	410 (32.69)	328 (39.39)	258 (31.07)	249 (30.39)	
Quartile 3 (60.68-97.67)	1,238 (33.95)	266 (21.07)	189 (21.82)	341 (40.12)	442 (55.38)	
Total bilirubin, µmol/L	11.98 ± 0.13	11.69 ± 0.25	11.98 ± 0.22	12.27 ± 0.24	12.08 ± 0.19	0.32
Total creatinine, µmol/L	99.37 ± 1.18	99.69 ± 1.52	103.27 ± 3.16	99.92 ± 3.00	95.26 ± 1.45	0.03
Total triglycerides, mmol/L	1.95 ± 0.05	1.95 ± 0.05	1.94 ± 0.06	1.97 ± 0.08	2.01 ± 0.12	0.97
CKD stage of eGFR, n (%)						0.11
G1-G2	1,610(46.06)	554 (48.15)	344 (43.79)	342 (44.90)	370 (46.53)	
G3a-G3b	1,846 (40.03)	600 (47.40)	411 (51.30)	426(50.93)	409 (51.24)	
G4-G5	190 (3.92)	69 (4.46)	50 (4.91)	42 (4.17)	29 (2.23)	
Total serum uric acid, µmol/L	354.60 ± 2.21	357.84 ± 3.58	354.46 ± 4.44	352.92 ± 3.95	352.41 ± 4.47	0.68
High density lipoprotein cholesterol, mmol/L	1.39 ± 0.01	1.35 ± 0.02	1.39 ± 0.02	1.38 ± 0.02	1.46 ± 0.03	<0.001
Alanine Aminotransferase, U/L	24.58 ± 0.94	23.76 ± 0.54	23.51 ± 0.71	27.38 ± 3.84	23.92 ± 0.50	0.78
Aspartate Aminotransferase, U/L	26.57 ± 0.39	26.75 ± 0.89	26.76 ± 0.80	26.82 ± 1.03	25.96 ± 0.40	0.66
Glycated hemoglobin (HbA1c), g/dL	5.99 ± 0.03	6.04 ± 0.04	6.00 ± 0.06	5.99 ± 0.05	5.93 ± 0.05	0.20

^a^These results are presented as [mean ± standard deviation, number (percentage)].

Abbreviation: CKD: Chronic Kidney Disease; eGFR: estimated Glomerular Filtration Rate.

During the follow-up period, a total of 1,593 deaths occurred, including 478 due to CVD and 268 attributable to cancer (Table 3). Covariate-adjusted analysis revealed that the HR for CKD patients in the highest intake group (quartile 4) was 0.74 (95% CI: 0.62-0.90) ((P_trend_ < 0.001). Likewise, the HR for cardiovascular disease mortality was 0.63 (95% CI: 0.45-0.88) (P_trend_ = 0.011), and for cancer-related mortality, it was 0.80 (95% CI: 0.52-1.23) ((P_trend_ = 0.176), when compared to the lowest intake group (quartile 1).

**Table 3.** The association of live microbe intake with all-cause, cardiovascular disease, and cancer mortality in chronic kidney disease patients.

	Dietary intake of MedHi live microbes^a^				P for trend
	Quartile 1	Quartile 2	Quartile 3	Quartile 4	
All-cause mortality					
No. death/total	559/1,593	402/1593	360/1592	272/1592	
Crude model	Ref.	0.91 (0.75, 1.09) 0.31	0.86 (0.73, 1.01) 0.07	0.66 (0.55, 0.80) <0.0001	<0.0001
Model 1	Ref.	0.89 (0.76, 1.05) 0.16	0.78 (0.68, 0.90) <0.001	0.68 (0.56, 0.81) <0.0001	<0.0001
Model 2	Ref.	0.88 (0.75, 1.04) 0.13	0.80 (0.69, 0.94) 0.01	0.74 (0.62, 0.90) <0.0001	<0.001
CVD mortality					
No. death/total	168/478	124/478	107/478	79/478	
Crude model	Ref.	0.86 (0.61, 1.21) 0.40	0.85 (0.63, 1.13) 0.27	0.60 (0.44, 0.82) 0.001	0.002
Model 1	Ref.	0.79 (0.59, 1.08) 0.14	0.76 (0.57, 0.98) 0.03	0.57 (0.42, 0.78) <0.001	<0.001
Model 2	Ref.	0.76 (0.57, 1.01) 0.06	0.78 (0.58, 1.06) 0.12	0.63 (0.45, 0.88) <0.0001	0.011
Cancer mortality					
No. death/total	95/268	70/268	56/268	47/268	
Crude model	Ref.	1.16 (0.81, 1.67) 0.40	0.92 (0.62, 1.37) 0.68	0.75 (0.51, 1.10) 0.14	0.068
Model 1	Ref.	1.08 (0.75, 1.57) 0.67	0.80 (0.56, 1.16) 0.24	0.66 (0.44, 0.98) 0.02	0.014
Model 2	Ref.	1.08 (0.76, 1.55) 0.66	0.81 (0.55, 1.21) 0.31	0.80 (0.52, 1.23) 0.32	0.176

^a^These results were presented as [OR value (confidence interval) P value].

Model 1 adjusted for age, gender, ethnicity, education, marital status and PIR;

Model 2 further adjusted for body mass index, serum triglycerides, serum uric acid, Alanine Aminotransferase, Aspartate Aminotransferase, creatinine, HbA1c (glycated hemoglobin), smoking, alcohol consumption, Healthy eating index (HEI-2015) score, metabolic equivalent of tasks, history of diabetes, hypertension and coronary heart disease.

Abbreviation: CVD: cardiovascular disease; Ref: reference; MedHi: medium and high live microbes.

The interaction analysis of all-cause mortality among CKD patients based on demographic characteristics were shown in Table 4. Statistical differences (*p* < 0.05) were observed between BMI groups and dietary live microbe intake in relation to all-cause mortality. Subgroup analysis suggested that participants aged ≥ 64, males, non-Hispanic blacks, those with a high school education level, widowed/divorced/separated marital status, PIR levels (1.1-3.0, >3.0) were associated with a decreased risk of mortality (all *p* < 0.05). In the analysis of CVD mortality, no interactions were observed across all covariates. Subgroup analysis indicated that decreased risk of cardiovascular death was associated with both females and males, the age group (≥78 years old), ethnicity (Mexican American, non-Hispanic white, and others), widowed/divorced/separated marital status, PIR level (>3.0). In the analysis of cancer mortality, interactions were found for age group and BMI (both *p* < 0.05). Subgroup analysis showed that PIR level (1.1-3.0) was associated with a decreased risk of cancer mortality (all *p* < 0.05). The interaction analysis of all-cause mortality among CKD patients based on clinical marks and chronic diseases characteristics were shown in Table 5. Statistical differences (*p* < 0.05) were observed between diabetes groups and dietary live microbe intake in relation to all-cause mortality. Subgroup analysis suggested that participants who have diabetes, hypertension, coronary heart disease and lower ALT levels (<17.00 U/L), AST levels and HbA1c levels (<5.5%) were associated with a decreased risk of mortality (all *p* < 0.05). In the analysis of CVD mortality, no interactions were observed across all covariates. Subgroup analysis indicated that decreased risk of cardiovascular death was associated with diabetes status, coronary heart disease, higher serum creatinine and HbA1c levels (all *p* < 0.05). In the analysis of cancer mortality, no interactions were found across all the covariates. Subgroup analysis indicated that decreased risk of cancer death was associated with coronary heart disease, lower HEI-2015 scores and higher serum creatinine groups (all *p* < 0.05).

**Table 4.** Subgroup analysis and interaction tests based on demographic of chronic kidney disease patients.

Variable	All-cause mortality			CVD mortality			Cancer-related mortality		
	HR (95% CI)^a^	P value	P for interaction	HR (95% CI)^a^	P value	P for interaction	HR (95% CI)^a^	P value	P for interaction
Gender			0.50			0.93			0.83
Female	0.92 (0.85, 1.01)	0.08		0.86 (0.76, 0.99)	0.03		0.93 (0.75, 1.15)	0.51	
Male	0.86 (0.79, 0.94)	0.001		0.82 (0.69, 0.96)	0.02		0.92 (0.77, 1.09)	0.31	
Age(years)			0.95			0.70			0.02
Quartile 1 (18-63)	0.93 (0.76, 1.12)	0.43		0.75 (0.48, 1.17)	0.20		1.32 (0.98, 1.79)	0.07	
Quartile 2 (64-77)	0.88 (0.79, 0.99)	0.03		0.94 (0.78, 1.13)	0.51		0.89 (0.72, 1.11)	0.30	
Quartile 3 (≥78)	0.91 (0.84, 0.97)	0.01		0.87 (0.76, 0.98)	0.02		0.77 (0.65, 0.93)		
Ethnicity			0.64			0.14			0.83
Mexican American	0.99 (0.78, 1.24)	0.90		1.62 (1.00, 2.61)	0.05		0.75 (0.39, 1.44)	0.39	
Non-Hispanic white	0.98 (0.83, 1.15)	0.80		0.86 (0.76, 0.98)	0.02		0.90 (0.78, 1.04)	0.14	
Non-Hispanic black	0.89 (0.83, 0.96)	0.001		0.87 (0.66, 1.14)	0.30		0.92 (0.64, 1.32)	0.65	
Others	0.79 (0.56,1.13)	0.20		0.43 (0.31, 0.59)	<0.001		0.55 (0.12, 2.41)	0.55	
Education			0.99			0.61			0.94
Below high school	0.95 (0.83, 1.09)	0.48		0.82 (0.61, 1.10)	0.18		1.14 (0.81, 1.61)	0.45	
High school	0.84 (0.77, 0.93)	<0.001		0.87 (0.74, 1.03)	0.10		0.82 (0.67, 1.00)	0.05	
Above high school	0.96 (0.86, 1.06)	0.39		0.90 (0.76, 1.06)	0.20		0.95 (0.78, 1.16)		
Marital status			0.49			0.54			0.43
Never married	0.87 (0.57, 1.33)	0.52		1.42 (0.60, 3.36)	0.42		0.78 (0.37, 1.64)	0.51	
Widowed/divorced/separated	0.86 (0.79, 0.93)	<0.001		0.84 (0.73, 0.98)	0.02		0.84 (0.68, 1.05)	0.13	
Married/living with partner	0.93 (0.86, 1.01)	0.09		0.91 (0.77, 1.07)	0.24		1.05 (0.87, 1.26)	0.61	
Poverty-income ratio			0.92			0.15			0.11
≤1	0.87 (0.73, 1.04)	0.12		0.85 (0.63, 1.15)	0.29		0.90 (0.60, 1.34)	0.60	
1.1-3.0	0.92 (0.86, 0.99)	0.04		0.94 (0.82, 1.09)	0.41		0.82 (0.68, 0.99)	0.04	
>3.0	0.88 (0.79, 0.98)	0.02		0.75 (0.61, 0.91)	0.005		1.11 (0.89, 1.37)	0.35	
Smoking status			0.69			0.09			0.42
Former	0.90 (0.83, 0.97)	0.01		0.92 (0.78, 1.10)	0.38		0.83 (0.69, 1.00)	0.05	
Never	0.89 (0.80, 0.99)	0.04		0.81 (0.69, 0.94)	0.01		1.00 (0.81, 1.22)	0.99	
Now	0.90 (0.73, 1.10)	0.30		0.93 (0.55, 1.58)	0.79		0.97 (0.65, 1.44)	0.87	
Drinking status			0.25			0.16			0.48
Never	0.77 (0.66, 0.89)	<0.001		0.66 (0.52, 0.84)	<0.001		0.92 (0.68, 1.24)	0.56	
Former	0.93 (0.84, 1.04)	0.20		0.87 (0.72, 1.05)	0.16		0.83 (0.66, 1.04)	0.11	
Mild	0.93 (0.84, 1.03)	0.14		0.91 (0.75, 1.10)	0.32		0.93 (0.74, 1.16)	0.52	
Moderate	1.19 (0.91, 1.56)	0.21		1.05 (0.49, 2.22)	0.90		1.20 (0.74, 1.95)	0.46	
Heavy	1.77 (0.56, 2.94)	0.09		1.27 (1.01, 1.55)	0.01		0.92 (0.48, 1.75)	0.79	
Body mass index			0.03			0.45			0.03
< 25.0	0.83 (0.74, 0.93)	0.001		0.85 (0.68, 1.07)	0.17		0.80 (0.62, 1.04)	0.09	
25.0-29.9	0.86 (0.77, 0.95)	0.004		0.80 (0.66, 0.97)	0.03		0.90 (0.74, 1.10)	0.32	
>29.9	1.97 (0.87, 3.01)	0.60		0.93 (0.77, 1.12)	0.45		1.06 (0.86, 1.32)	0.57	
Physical activity (MET)			0.85			0.84			0.99
Low	1.00 (0.83, 1.20)	0.98		0.93 (0.82, 1.05)	0.22		0.93 (0.77, 1.11)	0.40	
Middle	0.90 (0.84, 0.96)	0.003		0.74 (0.57, 0.95)	0.02		0.91 (0.70, 1.19)	0.50	
High	0.86 (0.77, 0.97)	0.01		1.00 (0.76, 1.33)	0.98		0.99 (0.69, 1.42)	0.94	

^a^These results are presented as [HR value (95% confidence interval)].

Abbreviation: CVD: cardiovascular disease; HR: hazard ratio; MET: metabolic equivalent of task.

**Table 5.** Subgroup analysis and interaction tests based on clinical marks and chronic diseases status of chronic kidney disease patients.

Variable	All-cause mortality			CVD mortality			Cancer-related mortality		
	HR (95% CI)^a^	P value	P for interaction	HR (95% CI)^a^	P value	P for interaction	HR (95% CI)^a^	P value	P for interaction
Diabetes			0.04			0.55			0.66
No	0.96 (0.88, 1.05)	0.39		0.91 (0.76, 1.09)	0.30		0.85 (0.68, 1.06)	0.15	
Yes	0.87 (0.80, 0.94)	<0.001		0.86 (0.75, 0.99)	0.04		0.96 (0.82, 1.13)	0.60	
Hypertension			0.67			0.87			0.82
No	0.94 (0.81, 1.11)	0.48		0.85 (0.60, 1.22)	0.39		1.08 (0.77, 1.51)	0.67	
Yes	0.88 (0.83, 0.95)	<0.001		0.84 (0.75, 0.95)	0.005		0.89 (0.76, 1.03)	0.10	
Coronary heart disease			0.19			0.13			0.07
No	0.92 (0.86, 0.98)	0.01		0.91 (0.80, 1.02)	0.11		0.99 (0.86, 1.12)	0.83	
Yes	0.79 (0.69, 0.90)	<0.001		0.68 (0.54, 0.85)	<0.001		0.60 (0.40, 0.89)	0.01	
Healthy eating index-2015 score			0.65			0.11			0.06
Quartile 1(<48.48)	0.88 (0.79, 0.99)	0.04		0.99 (0.83, 1.18)	0.89		0.77 (0.61, 0.97)	0.03	
Quartile 2 (48.48 ∼ 60.68)	0.91 (0.83, 1.01)	0.07		0.96 (0.80, 1.16)	0.69		0.91 (0.67, 1.22)	0.51	
Quartile (>60.68)	0.92 (0.85, 1.01)	0.09		0.79 (0.67, 0.93)	0.005		0.94 (0.73, 1.17)	0.21	
Serum triglycerides, mmol/L			0.20			0.93			0.06
Quartile 1(<1.21)	0.86 (0.79, 0.95)	0.002		0.86 (0.72, 1.03)	0.10		0.79 (0.62, 0.96)	0.02	
Quartile 2(1.21-2.02)	0.95 (0.85, 1.06)	0.36		0.93 (0.77, 1.12)	0.44		1.03 (0.81, 1.31)	0.81	
Quartile 3(>2.02)	0.88 (0.80, 0.98)	0.02		0.88 (0.72, 1.07)	0.19		0.98 (0.80, 1.19)	0.82	
Serum uric acid, µmol/L			0.59			0.46			0.70
Quartile 1(<309.31)	0.91 (0.82, 1.01)	0.08		0.92 (0.76, 1.11)	0.38		0.87 (0.68, 1.11)	0.25	
Quartile 2(309.31-392.64)	0.91 (0.83, 1.00)	0.06		0.87 (0.73, 1.04)	0.13		1.01 (0.82, 1.24)	0.95	
Quartile 3(>392.64)	0.89 (0.81, 0.97)	0.01		0.89 (0.75, 1.06)	0.19		0.85 (0.67, 1.08)	0.18	
Serum creatinine, µmol/L			0.63			0.80			0.34
Quartile 1(<81.33)	0.86 (0.76, 0.98)	0.02		0.72 (0.60, 0.88)	<0.001		1.00 (0.77, 1.30)	0.97	
Quartile 2(81.33-106.11)	0.98 (0.89, 1.09)	0.74		1.06 (0.87, 1.28)	0.57		1.02 (0.84, 1.23)	0.87	
Quartile 3(>106.11)	0.86 (0.79, 0.94)	0.86		0.84 (0.70, 0.94)	0.01		0.74 (0.59, 0.92)	0.01	
Aspartate aminotransferase, U/L			0.54			0.70			0.43
Quartile 1(<21.00)	0.90 (0.80, 1.02)	0.10		0.93 (0.78, 1.12)	0.46		1.02 (0.81, 1.29)	0.86	
Quartile 2(21.00-26.04)	0.90 (0.82, 0.99)	0.03		0.87 (0.74, 1.02)	0.08		0.95 (0.78, 1.15)	0.57	
Quartile 3(>26.04)	0.88 (0.79, 0.98)	0.02		0.84 (0.71, 0.99)	0.04		0.85 (0.67, 1.07)	0.17	
Alanine aminotransferase, U/L			0.53			0.92			0.07
Quartile 1(<17.00)	0.88 (0.81, 0.96)	0.003		0.90 (0.75, 1.08)	0.27		0.81 (0.68, 0.97)	0.02	
Quartile 2(17.00-23.14)	0.92 (0.85, 1.00)	0.05		0.82 (0.70, 0.97)	0.02		0.90 (0.72, 1.12)	0.34	
Quartile 3(>23.14)	0.90 (0.79, 1.03)	0.12		0.89 (0.75, 1.06)	0.20		1.00 (0.75, 1.33)	0.98	
Glycated hemoglobin (HbA1c), %			0.53			0.61			0.79
Quartile 1(<5.50)	0.89 (0.80, 0.99)	0.04		0.87 (0.77, 1.00)	0.04		1.03 (0.84, 1.26)	0.80	
Quartile 2(5.50-6.00)	0.89 (0.80, 0.98)	0.02		0.92 (0.74, 1.13)	0.41		0.87 (0.69, 1.10)	0.24	
Quartile 3(>6.00)	0.92 (0.83, 1.01)	0.08		0.80 (0.66, 0.98)	0.03		0.85 (0.67, 1.08)	0.19	

^a^These results are presented as [HR value (confidence interval)].

Abbreviation: CVD: cardiovascular disease; HR: hazard ratio; MET: metabolic equivalent of task.

**Figure 2.**
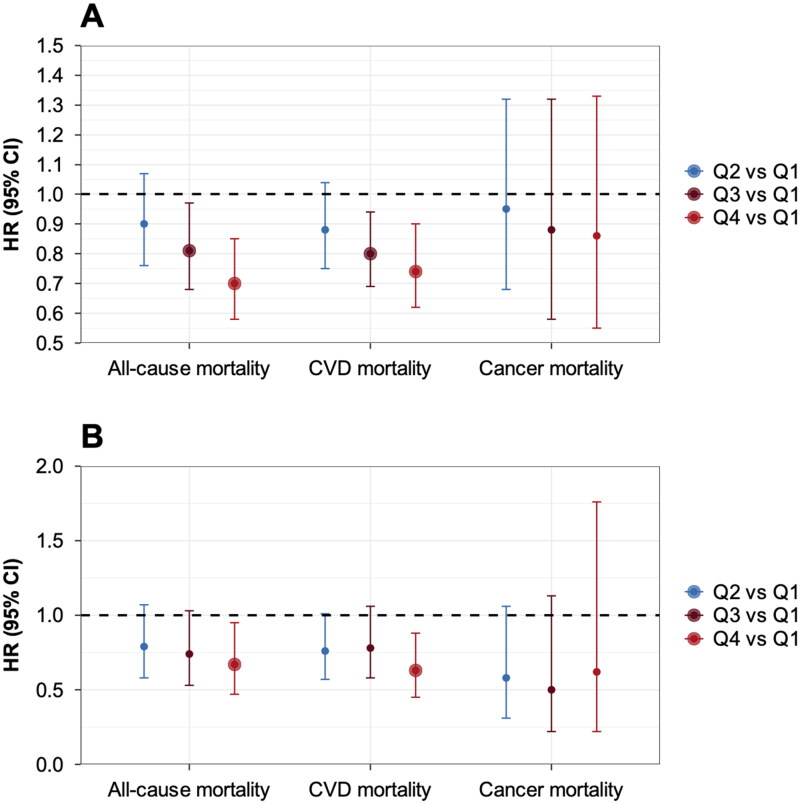
Sensitivity analyses were conducted, excluding: (A) participants with death outcomes in the first two years of follow-up, (B) CKD patients who consumed non-food probiotic and non-food prebiotics. Abbreviation: CKD: Chronic Kidney Disease; CVD: cardiovascular disease.

After excluding participants with death outcomes within two years of follow-up (Figure 2(A)), the HR for all-cause mortality in the highest intake group (quartile 4) was 0.70 (95% CI: 0.58-0.85) (*p* < 0.001), the quartile 3 group was 0.81 (95% CI: 0.68-0.97, *p* = 0.02) and the quartile 2 group was 0.90 (95% CI: 0.76-1.07, *p* = 0.23); the HR for CVD mortality in the highest intake group (quartile 4) was 0.74 (95% CI: 0.62-0.90) (*p* = 0.002), the quartile 3 group was 0.80 (95% CI: 0.69-0.94, *p* = 0.01) and the quartile 2 group was 0.88 (95% CI: 0.75-1.04, *p* = 0.13); the HR for cancer mortality in the highest intake group (quartile 4) was 0.86 (95% CI: 0.55-1.33) (*p* = 0.49), the quartile 3 group was 0.88 (95% CI: 0.58-1.32, *p* = 0.01) and the quartile 2 group was 0.95 (95% CI: 0.68-1.32, *p* = 0.75). After excluding participants with consumption of non-food probiotic and non-food prebiotics (Figure 2(B)), the HR for all-cause mortality in the highest intake group (quartile 4) was 0.67 (95% CI: 0.47, 0.95) (*p* = 0.03), the quartile 3 group was 0.74 (95% CI: 0.53-1.03, *p* = 0.07) and the quartile 2 group was 0.79 (95% CI: 0.58-1.07, *p* = 0.13); the HR for CVD mortality in the highest intake group (quartile 4) was 0.63 (95% CI: 0.45-0.88, *p* = 0.01), the quartile 3 group was 0.78 (95% CI: 0.58-1.06, *p* = 0.12) and the quartile 2 group was 0.76 (95% CI: 0.57-1.01, *p* = 0.06); the HR for cancer mortality in the highest intake group (quartile 4) was 0.62 (95% CI: 0.22-1.76, *p* = 0.37), the quartile 3 group was 0.50 (95% CI: 0.22-1.13, *p* = 0.10) and the quartile 2 group was 0.58 (95% CI: 0.31-1.06, *p* = 0.08). After excluding participants with using of antibiotics (Supplementary Table 2), the HR for all-cause mortality in the highest intake group (quartile 4) was 0.71 (95% CI: 0.58, 0.87), the quartile 3 group was 0.83 (95% CI: 0.70-0.98) and the quartile 2 group was 0.88 (95% CI: 0.74-1.04); the HR for CVD mortality in the highest intake group (quartile 4) was 0.60 (95% CI: 0.44-0.83), the quartile 3 group was 0.85 (95% CI: 0.60-1.19) and the quartile 2 group was 0.81 (95% CI: 0.60-1.08, *p* = 0.15); and the HR for cancer mortality in the highest intake group (quartile 4) was 0.76 (95% CI: 0.48-1.20), the quartile 3 group was 0.82 (95% CI: 0.55-1.21) and the quartile 2 group was 1.09 (95% CI: 0.77-1.55). The distribution of cardiovascular, metabolic, and other chronic disease medications used in the past year among CKD patients was presented in Supplementary Table 2. Additionally, we incorporated these medications as covariates in the study for sensitivity analysis. The results demonstrated that our primary findings remained robust, as shown in Supplementary Table 3.

## Discussion

To our knowledge, this is the first prospective study to explore the relationship between intake of food items rich in medium and high categories live microbes and all-cause and related cause mortality outcomes in CKD patients in the United States population. Our findings indicated that a high intake of food containing dietary live microbes was linked to a 26% reduced risk of all-cause mortality and a 37% reduced risk of CVD mortality in CKD patients. Additionally, a dose-response effect was observed. And the results of sensitivity analysis and stratified analysis were generally consistent.

Previous studies have reported the association between the intake of dietary living microbes and chronic diseases such as nonalcoholic fatty liver disease (NAFLD) [27], diabetic kidney disease [13] and abdominal aortic calcification [6]. These literatures indicated that dietary live microbes had a significant protective effect on these diseases. Dietary live microbes, especially those found in medium and high categories foods, included fermented foods such as vegetables, fruits, yogurt, and kimchi, the beneficial microbial communities in these foods have been studied for their protective effects on health [28,29]. Our study found that individuals with higher education levels tended to consume higher amounts of dietary live microbes. Some studies have shown that residents with a high level of education are more likely to adopt and cultivate healthy dietary patterns, thereby increasing their intake of dietary live microbes such as yogurt and prebiotics. Foods like yogurt and prebiotics are recognized for their ability to regulate gastrointestinal function and promote homeostasis within the internal environment [30,31]. And it was observed across various subgroups that dietary microbiota can reduce the mortality rate in CKD patients, particularly among older age groups (over 64 years), males, highly educated individuals, and non-obese individuals (BMI < 30.0). As physical and metabolic functions decline, along with the prevalence of chronic diseases such as hypertension and diabetes, older adults increasingly focus on dietary regulation, leading to a higher consumption of foods rich in live microbes, such as fresh milk and yogurt [32]. Probiotics, along with certain dietary live microbes, are digested and absorbed by the human digestive tract, where they colonize the gastrointestinal system and synthesize B vitamins. These vitamins play a crucial role in regulating various health indicators, including obesity, blood glucose levels, and blood pressure [33,34].

Some current studies indicated that daily intake of microbes played an important role in maintaining the balance of human gut microbiota, and the imbalance of human gut microbiota may have adverse effects on the prognosis of CKD patients [35,36]. Deterioration of intestinal barrier function, coupled with alterations in gut microbiome composition, may result in systemic inflammation associated with CKD [37]. The diverse microorganisms residing in the human gastrointestinal tract synthesize various bioactive substances, including adiponectin, which can mitigate intestinal permeability and help maintain intestinal homeostasis [38]. Previous experimental studies have demonstrated that a reduction in serum adiponectin concentration accelerates the progression of CKD. This decrease in adiponectin levels enhances the expression of transforming growth factor-beta (TGF-β) and interleukin-4 (IL-4) in the glomerulus, thereby promoting renal fibrosis [39,40]. In animal experiments, adiponectin deficiency alone resulted in the loss of podocyte processes and the presence of albumin in mice [41]. Adiponectin knockout mice exhibit a normal podocyte structure and show no signs of proteinuria [42]. And it was found that after two months of dietary probiotic treatment, blood urea levels in patients with CKD stages 3-4 decreased by over 10% compared to baseline levels [43]. Yacoub et al. divided 6,853 patients with CKD stages 3-5 into two groups based on their yogurt consumption habits: the regular consumption group (≥3 times per week) and the rare consumption group (≤ 3 times per week) [44]. After three years of comparative study, it was found that the urinary protein excretion rate in the regular consumption group was lower than that in the rare consumption group. Liu et al. found that the prevalence of CKD was lower in individuals who consumed probiotics, prebiotics, or yogurt compared to those who did not [45]. Dietary probiotics, probiotics or yogurt could reduce the risk of moderate CKD and high CKD by 12% and 11%, respectively. Meanwhile, some observational studies have also found that for CKD patients, taking 9 × 10^10^ CFU of *lactobacillus* daily for 6 consecutive months resulted in a decrease in blood urea nitrogen levels [46]. However, for CKD patients undergoing hemodialysis, the administration of 1.8 × 10^11^ CFU of probiotics daily for two consecutive months did not result in a decrease in blood uremic toxins. This may be attributed to the short duration of the trial and the relatively low dosage of probiotics. Furthermore, the metabolism of probiotics produces indoxyl glucuronide, a compound associated with gut microbiota imbalance, which cannot be effectively removed through hemodialysis therapy [47].

These survey and research indicated that the intake of dietary live microbes had a positive effect on regulating renal function indicators such as blood urea and urine protein. The homeostasis of the gut microbiota in the human body plays an important role in clearing endotoxins and maintaining immune function [48,49]. As for the CKD patients, supplementation with dietary live microbes can regulate the balance of gut microbiota. Live microbes from the diet can strongly adhere to intestinal mucosal epithelial cells *via* teichoic acid, creating a protective biological barrier that hinders the colonization and invasion of pathogenic bacteria. Meanwhile, products such as lactic acid produced by the metabolism of gut microbiota can lower the pH value of the intestine, effectively inhibit the growth of harmful bacteria and prevent the absorption of ammonia in the intestine, reduce the amount of urea involved in the liver intestinal cycle, and accelerate the excretion of toxic metabolites in the intestine [50].

In addition to creatinine and urea nitrogen, protein-bound toxins generated by gut microbiota also play a significant role in the progression of CKD. Guida et al. conducted a randomized, double-blind clinical trial involving 30 patients with end-stage renal disease. In this study, the experimental group received probiotics and prebiotics, while the control group was given a placebo. The results indicated a significant decrease in protein-bound uremic toxins (PCS) in the experimental group [51]. After targeted supplementation of bifidobacteria to hemodialysis patients, the concentration of IS also significantly decreased. IS and PCS can activate transforming growth factor by stimulating the renin angiotensin aldosterone system β Pathways exacerbate the progression of renal fibrosis in CKD patients [52]. The intake of live microbes through the human diet, by adjusting the balance of gut microbiota, can compete for nutrients with bacteria that produce indole-3-phenol and p-cresol, inhibit the growth of precursor bacteria that synthesize protein binding toxins, and thus reduce the content of protein binding toxins [53]. In addition, studies have also found that short chain fatty acids such as acetate, propionate, and butyrate, which were byproducts of the gut microbiota, improve acute renal dysfunction by reducing local and systemic inflammation, reducing cellular oxidative stress, and cell infiltration and apoptosis [54].

Several limitations are present in this study. Firstly, the intake of dietary live microbes in the surveyed population was collected through retrospective dietary surveys, inevitably leading to recall bias, and the justification of performing this research on intakes of live microbes is low. Secondly, drawing from baseline survey data, assessing the presence of live microbes in food can result in biases influenced by dietary changes over time. Thirdly, although adjustments have been made for potential confounding factors, there may still be additional confounding factors that affect the intake of live microbes and the health outcomes of CKD. Fourthly, due to the varied dietary habits among individuals with different dietary patterns, further research is needed to investigate the association between live microbes from different food sources and the health outcomes of CKD patients. Fifthly, since the NHANES database does not provide 16S or metagenomic sequencing data on the baseline microbiota status of the participants, we were unable to accurately assess the microbiota status of the participants. Finally, the population studied in this survey is the American population and the sample size is small; further exploration is needed to determine the association between dietary live microbes and CKD outcomes in other regions to validate its generalizability.

## Conclusion

Consuming a high amount of food items rich in medium and high categories of dietary live microbes can reduce both all-cause mortality and CVD mortality in CKD patients. This association strengthens with increasing intake of dietary live microbes. In the future, clinical trials and multicenter cohort studies in CKD patients should be considered to further evaluate the impact of medium and high categories of dietary live microbes on the near- and long-term adverse health outcomes of CKD patients.

## Supplementary Material

Conflict of Interest form.docx

Supplementary tables.docx

## Data Availability

Researchers can request the data used for the analysis from the NHANES (https://www.cdc.gov/nchs/nhanes/index.htm). The original contributions presented in the study are included in the article/supplementary material, further inquiries can be directed to the corresponding author.
