# Supplementary material for: Association of live microbes intake and risk of all-cause, cardiovascular disease, and cancer-related mortality in patients with chronic kidney disease
Source: Ren Fail. 2025 Jan 6;47(1):2449196. doi: 10.1080/0886022X.2024.2449196 (PMC11721851; doi:10.1080/0886022X.2024.2449196)
Supplement: Supplementary tables.docx [file IRNF_A_2449196_SM3905.docx]

**Supplementary table 1.** The distribution of cardiovascular, metabolic, and other chronic disease medications used in the past year among chronic kidney disease patients.

| Medicine categories | Dietary intake of MedHi live microbes ^a^ | | | | P value |
| --- | --- | --- | --- | --- | --- |
|  | Quartile 1 | Quartile 2 | Quartile 3 | Quartile 4 |  |
| Anti-diabetic drugs, n (%) ^b^ |  |  |  |  | 0.46 |
| Yes | 301 (21.84) | 205 (20.56) | 184 (20.15) | 189 (18.24) |  |
| No | 922 (78.16) | 605 (79.44) | 621 (79.85) | 619 (81.76) |  |
| Anti-hypertension drugs, n (%) ^c^ |  |  |  |  | 0.01 |
| Yes | 838 (63.48) | 532 (62.12) | 560 (64.17) | 516 (54.80) |  |
| No | 385 (36.52) | 278 (37.88) | 245 (35.83) | 292 (45.20) |  |
| Anti-hyperlipidemic drugs, n (%) ^d^ |  |  |  |  | 0.35 |
| Yes | 492 (36.79) | 303 (35.72) | 341 (39.87) | 312 (34.92) |  |
| No | 731 (63.21) | 507 (64.28) | 464 (60.13) | 496 (65.08) |  |
| Anti-metabolites drugs, n (%) ^e^ |  |  |  |  | 0.55 |
| Yes | 5 (0.47) | 4 (0.61) | 7 (0.69) | 1 (0.18) |  |
| No | 1,218 (1218) | 806 (99.39) | 798 (99.31) | 807 (99.82) |  |

^a^ The results are presented as number (percentage).

^b^ Anti-diabetic medications include chlorpropamide, glimepiride, glipizide, glyburide, various types of insulin (aspart, detemir, glargine, glulisine, isophane, lispro), linagliptin, liraglutide, metformin, miglitol, nateglinide, pioglitazone, repaglinide, rosiglitazone, sitagliptin, tolazamide, among others.

^c^ Anti-hypertension medications include acebutolol hydrochloride, amiloride, amlodipine (and amlodipine besylate), atenolol, benazepril (and benazepril hydrochloride), betaxolol (and betaxolol hydrochloride), bisoprolol (and bisoprolol fumarate), bumetanide, candesartan (and candesartan cilexetil), captopril, carvedilol, chlorothiazide, clonidine (and clonidine hydrochloride), diazoxide, diltiazem (and diltiazem hydrochloride), diuretics (unspecified), dorzolamide, doxazosin mesylate, enalapril (and enalapril maleate), felodipine, fluvastatin sodium, fosinopril, furosemide, guanfacine hydrochloride, hydrochlorothiazide, indapamide, irbesartan, labetalol (and labetalol hydrochloride), lisinopril, losartan (and losartan potassium), methazolamide, methyldopa, metoprolol (succinate and tartrate), moexipril (and moexipril hydrochloride), nadolol, nebivolol, nifedipine, nisoldipine, nitrofurantoin, nitroglycerin, olmesartan, penbutolol sulfate, pindolol, prazosin hydrochloride, propranolol (and propranolol hydrochloride), quinapril (and quinapril hydrochloride), ramipril, reserpine, sotalol (and sotalol hydrochloride), spironolactone, tamsulosin hydrochloride, telmisartan, terazosin hydrochloride, timolol maleate, torsemide, trandolapril, triamterene, valsartan, verapamil (and verapamil hydrochloride), and others.

^d^ Anti-hyperlipidemic medications include amlodipine, atorvastatin (and atorvastatin calcium), cerivastatin sodium, cholestyramine, colesevelam, colestipol, ezetimibe, fenofibrate, fenofibric acid, fluvastatin (and fluvastatin sodium), gemfibrozil, lovastatin, niacin, pitavastatin, pravastatin (and pravastatin sodium), rosuvastatin, simvastatin, and other unspecified antihyperlipidemic agents.

^e^ Anti-metabolites drugs: methotrexate, hydroxyurea, methotrexate sodium.

**Supplementary table 2**. The association of live microbe intake with all-cause, cardiovascular disease, and cancer mortality in chronic kidney disease patients who did not use antibiotics within one year.

|  | Dietary intake of MedHi live microbes ^a^ | | | | P for trend |
| --- | --- | --- | --- | --- | --- |
|  | Quartile 1 | Quartile 2 | Quartile 3 | Quartile 4 |  |
| All-cause mortality |  |  |  |  |  |
| No. death/total | 514/1474 | 344/1474 | 341/1474 | 275/1474 |  |
| Crude model | Ref. | 0.90 (0.74, 1.09) 0.29 | 0.88 (0.73, 1.05) 0.15 | 0.65 (0.54, 0.79) <0.0001 | <0.0001 |
| Model 1 | Ref. | 0.87 (0.74, 1.02) 0.10 | 0.78 (0.66, 0.91) 0.002 | 0.66 (0.54, 0.79) <0.0001 | <0.0001 |
| Model 2 | Ref. | 0.88 (0.74, 1.04) 0.13 | 0.83 (0.70, 0.98) 0.03 | 0.71 (0.58, 0.87) 0.001 | <0.001 |
| CVD mortality |  |  |  |  |  |
| No. death/total | 156/440 | 104/440 | 101/440 | 79/440 |  |
| Crude model | Ref. | 0.86 (0.61, 1.21) 0.39 | 0.86 (0.63, 1.17) 0.34 | 0.56 (0.41, 0.76) <0.001 | <0.001 |
| Model 1 | Ref. | 0.79 (0.59, 1.06) 0.12 | 0.77 (0.57, 1.04) 0.08 | 0.53 (0.39, 0.73) <0.0001 | <0.001 |
| Model 2 | Ref. | 0.81 (0.60, 1.08) 0.15 | 0.85 (0.60, 1.19) 0.33 | 0.60 (0.44, 0.83) 0.002 | 0.006 |
| Cancer mortality |  |  |  |  |  |
| No. death/total | 85/245 | 62/245 | 53/245 | 45/245 |  |
| Crude model | Ref. | 1.15 (0.79, 1.67) 0.48 | 0.92 (0.61, 1.39) 0.71 | 0.73 (0.49, 1.11) 0.14 | 0.070 |
| Model 1 | Ref. | 1.05 (0.72, 1.51) 0.81 | 0.78 (0.53, 1.13) 0.19 | 0.63 (0.40, 0.98) 0.04 | 0.011 |
| Model 2 | Ref. | 1.09 (0.77, 1.55) 0.63 | 0.82 (0.55, 1.21) 0.31 | 0.76 (0.48, 1.20) 0.24 | 0.124 |

^a^ The results are presented as HR value (95% confidence interval) P value.

Model 1 adjusted for age, gender, ethnicity, education, marital status and PIR (poverty income ratio);

Model 2 further adjusted for BMI (body mass index), serum triglycerides, serum uric acid, ALT (Alanine Aminotransferase), AST (Aspartate aminotransferase), creatinine, HbA1c (Glycated Hemoglobin), smoking, alcohol consumption, HEI-2015(Healthy eating index-2015) score, metabolic equivalent of tasks (MET), history of diabetes, hypertension and coronary heart disease.

Abbreviation: HR, hazard ratio; Ref: reference; MedHi, medium and high live microbes.

**Supplementary table 3.** The association of live microbe intake with all-cause, cardiovascular disease, and cancer mortality in chronic kidney disease patients was further adjusted for the use of drugs for cardiovascular, metabolic, and other chronic diseases.

|  | Dietary intake of MedHi live microbes ^a^ | | | | P for trend |
| --- | --- | --- | --- | --- | --- |
|  | Quartile 1 | Quartile 2 | Quartile 3 | Quartile 4 |  |
| All-cause mortality |  |  |  |  |  |
| No. death/total | 559/1593 | 402/1593 | 360/1592 | 272/1592 |  |
| Crude model | Ref. | 0.89 (0.74, 1.08) 0.23 | 0.87 (0.74, 1.03) 0.11 | 0.66 (0.55, 0.80) <0.0001 | <0.0001 |
| Model 1 | Ref. | 0.89 (0.76, 1.04) 0.14 | 0.79 (0.68, 0.91) 0.001 | 0.68 (0.56, 0.81) <0.0001 | <0.0001 |
| Model 2 | Ref. | 0.90 (0.76, 1.05) 0.19 | 0.85 (0.73, 0.98) 0.03 | 0.75 (0.62, 0.91) 0.004 | 0.002 |
| CVD mortality |  |  |  |  |  |
| No. death/total | 168/478 | 124/478 | 107/478 | 79/478 |  |
| Crude model | Ref. | 0.85 (0.61, 1.19) 0.34 | 0.86 (0.64, 1.16) 0.32 | 0.60 (0.44, 0.82) 0.001 | 0.003 |
| Model 1 | Ref. | 0.79 (0.59, 1.07) 0.13 | 0.76 (0.58, 1.00) 0.05 | 0.57 (0.42, 0.78) <0.001 | <0.001 |
| Model 2 | Ref. | 0.79 (0.59, 1.06) 0.12 | 0.82 (0.60, 1.11) 0.19 | 0.65 (0.47, 0.91) 0.01 | 0.021 |
| Cancer mortality |  |  |  |  |  |
| No. death/total | 95/268 | 70/268 | 56/268 | 47/268 |  |
| Crude model | Ref. | 1.14 (0.80, 1.62) 0.48 | 0.94 (0.64, 1.39) 0.76 | 0.75 (0.51, 1.10) 0.14 | 0.078 |
| Model 1 | Ref. | 1.07 (0.74, 1.55) 0.71 | 0.81 (0.57, 1.17) 0.26 | 0.66 (0.44, 1.00) 0.05 | 0.015 |
| Model 2 | Ref. | 1.06 (0.73, 1.52) 0.77 | 0.86 (0.59, 1.24) 0.42 | 0.78 (0.51, 1.19) 0.25 | 0.162 |

^a^ The results are presented as HR value (95% confidence interval) P value.

Model 1 adjusted for age, gender, ethnicity, education, marital status and PIR;

Model 2 further adjusted for BMI, serum triglycerides, serum uric acid, ALT, AST, creatinine, HbA1c, smoking, alcohol consumption, HEI-2015 score, metabolic equivalent of tasks (MET), history of diabetes, hypertension and coronary heart disease.

Abbreviation: HR, hazard ratio; Ref: reference; MedHi, medium and high live microbes.
